# Development of Inoculants for Aluminum Alloy: A Review

**DOI:** 10.3390/ma16155500

**Published:** 2023-08-07

**Authors:** Shuiqing Liu, Tong Zhao, Jinyuan Fu, Qun Zu

**Affiliations:** 1 State Key Laboratory for Reliability and Intelligence of Electrical Equipment, Hebei University of Technology, Tianjin 300401, China; 2 School of Mechanical Engineering, Hebei University of Technology, Tianjin 300401, China; fjy18238941292@163.com; 3 Arizona College of Technology, Hebei University of Technology, Tianjin 300401, China; zhaotong20022021@163.com

**Keywords:** inoculate, grain refinement, Al-Ti-B, Al-Sc alloy, Fe-rich phase in Al-Si alloy

## Abstract

Aluminum and its alloys are widely used in packaging, transportation, electrical materials, and many other fields because of their abundance, light weight, good mechanical properties, suitable corrosion resistance, excellent electrical conductivity, and other advantages. Grain refinement achieved by adding inoculant is important not only to reduce the segregation and thermal cracking of alloy castings but also to improve the mechanical properties of alloy castings. Therefore, fine equiaxed grain structure has always been one of the goals pursued by the aluminum alloy casting industry. For this reason, the selection and development of effective inoculants for aluminum alloy is a key technology in the aluminum processing industry. This paper summarizes the development history of inoculants for aluminum alloy, including Al-Ti-C, Al-Ti-B, Al-Ti, Al-Ti-B-(C)-Ce, Al-Sc, and the Fe-rich phase of Al-Si alloy. At the same time, the advantages and disadvantages of common inoculants are introduced and prospective future applications are reviewed.

## 1. Introduction

In the aluminum alloy industry, grain refinement is often used to improve the strength and plasticity of the material, and for grain sizes of the product above the nano level, refining the grain can improve the dislocation density and increase the strength of the material. Compared with large grains, small, coordinated deformation per unit volume can improve the extension of the overall material and increase the plasticity of the material [1]. Grain refinement can also reduce the twinning caused by dendrite growth, reduce the tendency for segregation, cold isolation, and hot cracking in the process, improve the feeding during solidification, eliminate or better disperse loose grains, improve the air tightness and surface quality of the casting, and improve the casting success rate [2,3,4]. 

Grain refinement refers to the grain transformation process that inhibits the growth of columnar grains and promotes the formation of fine equiaxed grains in castings. Therefore, in order to expand the equiaxed grains region and reduce the columnar grains region, inoculants can be added to the aluminum alloy to promote the transformation of grain from columnar to equiaxed. According to the basic theory of material science, the methods of grain refinement can be classified into two major categories: physical methods and chemical methods. Physical methods include rapid cooling, physical field refinement, and mechanical-physical refinement. On the other hand, chemical methods involve the addition of grain refiners, which promote grain nucleation or hinder the growth of crystals to achieve fine-grained structures. Currently, adding inoculants to aluminum melts is considered the most effective and practical grain refinement method due to its fast action, excellent refining results, easy operation, and adaptability. The inoculants are mainly added in the form of aluminum base intermediate alloys. At present, grain refiners are mainly added in the form of aluminum base intermediate alloys. The existing common refiners mainly include Al-Ti-B, Al-Ti-C, Al-Ti-B-C, Al-B-Ce, Al-Sc, and Al-Sc-Zr; among which, Al-Ti-B wire is the most widely used in industry. The grain refiner used by more than 80% of aluminum processing enterprises in the world is Al-Ti-B wire intermediate alloy.

In 1930, pure Ti was added to aluminum ingots and then aluminum grains were refined [5]. With the development of grain-refining technology, mixed salts such as K_2_TiF_6_ and KBF_4_ have been gradually applied to grain refining of aluminum alloys, but they produce a large amount of toxic fluoride gas and slag inclusion and have unstable refining efficiency [6,7,8]. In the 1960s, the era of intermediate alloys was entered. With the emergence and promotion of coreless induction furnaces, the Al-Ti binary alloy was first developed, and then a more effective Al-Ti-B intermediate alloy was successfully developed. The stability of its refinement effect was greatly improved by the addition of B element. At the beginning, Al-Ti-B is added in bulk. Although it can play a good thinning role, the second-phase particle density is large, which is easy to gather and precipitate during static so that the thinning effect declines, and it is “poisoned” when it is subjected to elements such as Zr. With the development of semicontinuous and continuous casting, by the mid-1970s, the United States developed a new Al-Ti-B refining technology, that is, in the form of a wirefeeder continuously added to the flow tank melt, not only to achieve the automation of refining processing, but also to effectively reduce the agglomeration of the second-phase particles. After the 1980s, various Al-Ti-B intermediate alloys were developed, of which Al-5Ti-1B intermediate alloy was the most widely used. In 1985, German researcher Banerji [9,10,11] and others developed Al-Ti-C intermediate alloy, which opened up a new field for grain refiners. Since the 1990s, the development of a new series of refiners, including Al-Ti-C-B, Al-Ti-B-Re, and Al-Ti-C-Re, has achieved certain results.

## 2. Specific Grain Refiner

### 2.1. Al-Ti-B

Al-Ti-B grain inoculant has a good refining effect and has a wide range of applications. It is the most important refiner, with remarkable refining effect in the aluminum alloy casting industry. The advantages of Al-Ti-B are its clear thinning effect, good stability, and lower price. The disadvantage is that the refining behavior of Al-Ti-B refiner is significantly affected by many factors such as particle distribution, refining process, alloy composition, and cooling rate. 

At present, there are various theories on the refinement mechanism of Al-Ti-B, which complement each other. The main refinement theories include phase diagram theory, particle theory, dual nucleation theory, etc. The dual nucleation theory can explain the grain-refining process of Al-Ti-B grain refiner well. The phase diagram theory was first proposed by Crossley based on the Al-Ti binary phase diagram [12]. Peritectic reaction occurs at 0.15%Ti and 665 °C, which promotes α-Al nucleation and achieves grain refinement L + TiAl_3_→α-Al. Maxwell found that TiAl_3_ particles existed in the grain center and considered that peritectic reaction was the main refinement mechanism [13]. Some scholars have proposed new explanations and opposing views. Based on the controversy, the phase diagram theory is not adequate to correctly explain the mechanism of Al-Ti-B grain refinement [14,15,16]. The boride particle theory was first proposed by Cibula [6,7,8]. According to the theory, TiB_2_ has a high melting point and can become an effective nucleating particle in the melt. However, it is found that in the absence of abundant solutes, Ti in the melt, single TiB_2_ particles, and clusters of TiB_2_ particles in the Al matrix will be pushed to the grain boundary by α-Al dendrites, which makes it difficult for the particle to become an effective hetero-nucleation core of α-Al, and it cannot produce a grain-refinement effect [17]. Therefore, it is difficult for particle theory to perfectly explain the mechanism of Al-Ti-B grain refining. Combining the phase diagram theory with the particle theory, Mohanty proposed the dual nucleation theory to explain the grain refinement mechanism of Al-Ti-B [17]. Mohanty holds the view that TiB_2_ with a high melting point is relatively stable after entering the melt, while TiAl_3_ will dissolve quickly, and the dissolution of TiAl_3_ will bring excess Ti to local areas of the melt. There is a concentration gradient of Ti on the surface of TiB_2_, so Ti is easy to segregate on its surface, form a new layer of TiAl_3_ after supercooling, and react with Al peritectic to form crystal nuclei after the temperature drops. Backerud put forward the theory of a peritectic shell, but the theory is inconsistent with practice [18]. In recent years, many scholars have conducted research under the framework of the dual nucleation theory [19,20,21,22]. In summary, dual nucleation can explain the grain refinement mechanism of Al-Ti-B more correctly, which is of great research significance.

**Figure 1:**
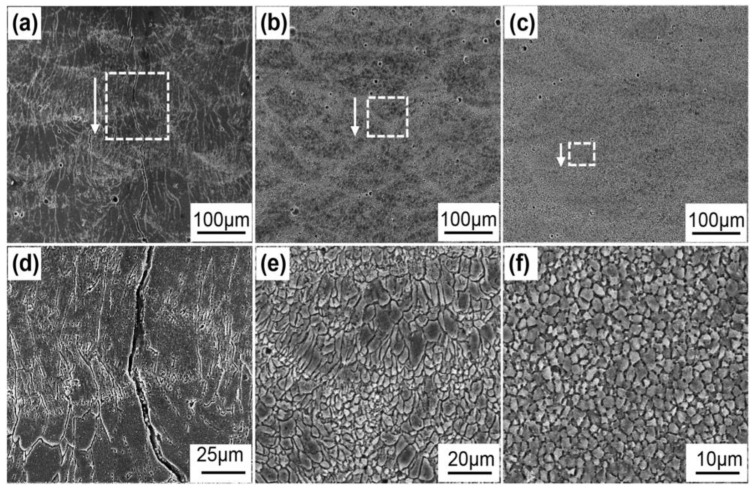
SEM micrograph of AA7075 Al alloy sample prepared by SLM using Al-Ti-B as grain refiner. (**a**) AA7075 sample without inoculant; (**b**) TiH2/AA7075 sample; (**c**) (TiH2+B)/AA7075 sample; (**d**–**f**) are the enlarged view of the rectangle region corresponding to (**a**–**c**) [39].

The preparation methods of Al-Ti-B refining agent include the high-temperature self-spreading method (high cost, not suitable for industrial production), thermite reduction method (not easy to operate, slow reaction, high energy consumption, and B recovery rate is only 25%), electrolysis method (difficult to produce intermediate alloys with high Ti and B content), and fluorine thermite reaction method (simple process, low cost, and suitable for industrial promotion). However, the size of the second phase, the recovery rate of B, and the uniformity of the organization are difficult to control, and the impurities are difficult to completely remove, requiring strict air process conditions [23,24]. The powder metallurgy rule can effectively control the size, distribution, and element ratio of the second phase in the refiner by changing the raw material and preparation process and is expected to achieve a better thinning effect [25,26,27,28,29]. In recent years, nano-ceramic particles, with their good thermal and chemical stability, have been increasingly used for aluminum and aluminum alloy grain refinement. Nanoparticles can provide rich nucleation particles for α-A, and some particles in the solidification process will be adsorbed to the solid–liquid interface, which will hinder the liquid phase of the molten atom to the growth interface diffusion and inhibit the grain growth [30,31,32,33]. However, the difficulty of such refiners in refining grains lies in the agglomeration phenomenon caused by the high surface energy of nanoparticles, and their poor wettability with aluminum melt leads to the rapid settlement of agglomeration in aluminum melt, which seriously weakens the refining effect. Improving the dispersion of nanoparticles in refiners is an important problem to be solved in grain refinement of this type of refiners, and various attempts, such as electrical pulse and ultrasonic vibration, have not obtained ideal results [34,35,36]. Recently, high-energy ball milling has been increasingly used to prepare micron metal/nano second-phase composite powder refiners [22,37,38]. The particle agglomeration phenomenon is effectively reduced, the dispersion of nanoparticles in Al melt is more uniform, and a better refinement effect is obtained. However, current studies mainly focus on the refinement effect and refinement mechanism, among which the key dispersion of nano second-phase particles and its influence on the refinement effect and mechanism are still unclear. Figure 1 shows the effect diagram of aluminum titanium-boron grain refiner prepared by the SLM (selective laser melting) method when it is actually applied to alloy refining.

The forming methods of Al-Ti-B wire mainly include the semicontinuous casting extrusion method (increased heat treatment process, low efficiency, high energy consumption, uneven composition and organization, and unstable product quality), continuous casting extrusion method (low energy consumption and stable product quality but slow casting speed and low production efficiency), continuous casting method, and continuous casting and rolling method (low energy consumption, high production efficiency, and product quality is stable). Extrusion casting, also known as liquid metal forging, is one of the effective methods of grain refinement. Extrusion casting is a casting and forging combined production process; that is, liquid metal is formed under high pressure and accompanied by plastic deformation so that the grain inside the material is broken and refined, and the defect density is reduced, thereby improving the performance of the material. Extrusion casting has a wide range of applications, including in automotive, high-speed rail, communications, and other fields. Compared with traditional die casting, extrusion casting has fewer defects such as pores and shrinkage holes, and the internal organization of the product is tighter and more uniform. At the same time, the extrusion casting process is suitable for large-scale operation, mechanized operation, and short production cycles.

**Figure 2:**
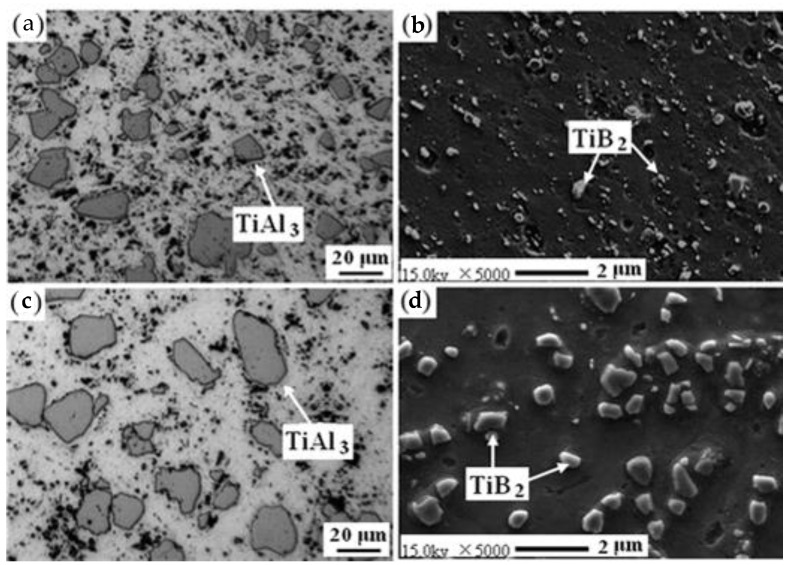
The Al-5Ti-1B ribbon and SEM image of its microstructure: (**a**) TiAl_3_ phase; (**b**,**d**) TiB_2_ phase; (**c**) TiAl_3_ phase [42].

It was found that the second phase (TiAl_3_ and TiB_2_) of the refiner is a good nucleating matrix of α-Al, which plays a role in refining the grain [40,41,42]. The grain-refining effect of Al-Ti-B refiner is mainly determined by the microstructure of the alloy, including the shape, size, and distribution of TiAl_3_ phase and TiB_2_ particles. As can be seen in Figure 2, the results show that the Al-5Ti-1B intermediate alloy is composed of Al matrix, TiB_2_, and a small amount of the TiAl_3_ phase. When the addition of TiB_2_ is constant, the grain size decreases with the increase in Al alloy solute content (i.e., the larger growth limiting factor Q). When the alloy composition is constant, the grain density increases, and the grain size decreases with the addition of TiB_2_. When there are a large number of dispersed and evenly distributed TiB_2_ and irregular lumps of TiAl_3_ in Al-Ti-B refiner, the refining effect is the best.

The more dispersed and smaller the nucleating particles in Al-Ti-B grain refiner, the stronger the grain-refining ability is. The microstructure of the refiner can be controlled in various ways. For example, a rapid solidification process can provide a large degree of supercooling, promote the nucleation of TiAl_3_ and TiB_2_, and reduce gravity segregation. The addition of ultrasonic and electromagnetic energy fields can stir the melt, disperse the nucleating particles, and break the dendrites, thus reducing the size of the nucleating particles. Therefore, certain methods can be used to homogenize the refiner structure and reduce the particle size of TiAl_3_ and TiB_2_, which is very beneficial to improve the grain-refining effect of Al-Ti-B.

The TiB_2_ particles are easy to aggregate and precipitate, which degrades the thinning effect. In the case of Zr, Cr, or Mn, B in TiB_2_ particles will be taken away, forming the corresponding borides, making TiB_2_ particles “poisoned”, and weakening or eliminating the thinning effect. On the contrary, Mg, Nb, and other elements can promote the grain-refining effect, and Mg can effectively inhibit the poisoning phenomenon of Al-5Ti-1B refiner caused by Si element. Therefore, revealing the influence of alloy composition on the refining ability of Al-Ti-B can clarify the applicability of Al-Ti-B refiner and avoid the increase in production cost. By adjusting the addition method, addition temperature, and retention time of Al-Ti-B refiner, the agglomerative precipitation phenomenon of TiB_2_ and the “poisoning” phenomenon of related elements can be effectively weakened. Some studies consider that the reaction of Zr with Si or impurity elements is the main factor causing toxicity [43].

However, Zr has a negative effect on the grain-refining ability of Al-Ti-B refiner [44], resulting in the grain-refining effect becoming worse and irreversible and the grains gradually coarsening with the extension of holding time, which is referred to as the Zr poisoning effect. Although there is no precise theory to explain the mechanism of Zr toxicity [44], it can be explained from two perspectives: (1) the influence of the reaction between Zr and TiB_2_ and TiAl_3_ on the nucleation ability of Al-Ti-B is studied. According to the nucleation theory, TiB_2_ and TiAl_3_ are important nucleating particles in Al-Ti-B refiner, and their existence states directly affect the grain-refining ability of the refiner. Therefore, the reaction between Zr and TiB_2_ and TiAl_3_ may reduce the refining ability of the refiner. According to Jones et al., this is because Zr replaces Ti in TiB_2_ and generates ZrB_2_ covering the surface of TiB_2_, which greatly weakens the nucleation ability of TiB_2_ and poisons the refiner. Abdel-Hamid considers that Zr may diffuse into TiB_2_, gradually forming a stable solid solution phase, thereby reducing the hetero-nucleation ability of TiB_2_. Murty holds the view that the reaction generates Al-Ti-Zr ternary compounds with weaker nucleation ability than TiB_2_. Bunn thinks that this toxic effect is due to Zr replacing Ti atoms in the TiAl_3_ layer on the surface of TiB_2_ [45]. Recent studies based on the dual nucleation theory suggest that the toxicity is caused by the replacement of Ti by Zr in the TiAl_3_ layer of TiB_2_ [46,47]. In addition, molecular dynamics simulation results show that the Ti_2_Zr_2_ compound roughens the surface of TiB_2_ and reduces the atomic order of the surface [48]. (2) The effect of the reaction between Zr and Ti or impurity elements on grain growth was studied. Spittle showed that adding trace elements such as Zr, Fe, Si, or Cr to pure Al melt alone could refine the grains [49,50]. However, when Zr and Fe or Zr and Si solute elements are present in the pure Al melt added to Al-Ti alloy, Zr will interact with other elements to form intermetallic phase, and the reduction of these solute elements will reduce the growth restriction effect of solute, resulting in grain coarsening. Johnsson considered that Zr, Ti, and Al formed ternary compounds, which reduced the Ti solute in the melt and the inhibitory effect of Ti solute on the growth of Al grains [51]. Qiu calculated the matching relationship between Al_8_Fe_4_Zr and Al substrate using the edge–edge matching model, indicating that Al_8_Fe_4_Zr reduces the nucleation ability of TiAl_3_ [52,53].

In forged aluminum alloys, the grain-refinement effect of Al-Ti-B is more significant due to the absent or low Si [54], but for the most widely used cast aluminum alloys, the high content of solute Si is prone to greatly attenuating the refining ability of Al-Ti-B and resulting in a toxic effect [55,56]. At present, it is widely recognized that TiB_2_ and TiAl_3_ particles in grain refiners interact with Si solves to form intermetallic compounds, which causes TiB_2_ to lose its nucleation ability and blocks the epitaxial nucleation of α-Al. The latest research shows that the cause of Si toxicity may be the segregation of Si solute at the TiB_2_/Al interface [57].

To sum up, Zr toxicity and Si toxicity are the major diseases restricting the thinning effect of Al-Ti-B. Although some researches have explained them, there is still a lack of a perfect unified theoretical mechanism. Recent studies on the toxic effect show that the reduction of nucleation efficiency of TiB_2_ is mainly related to the newly formed compounds with low nucleation ability on the surface of the upper Al_3_Ti layer (such as Ti_2_Zr, Al_8_Fe_4_Zr, TiSi, TiSi_2_, Ti_5_Si_3_, etc.). At the same time, it may also be caused by the expansion of the invisible nuclear zone during solidification. Based on the importance of Al-Ti-B refiner and its toxic effect, ultrasonic treatment with melt is expected to inhibit or eliminate the toxic effect so as to significantly improve the grain-refining technology level of aluminum alloy.

In order to improve the production conditions of aluminum alloy grain refiners and solve the problems of thinning decline and poisoning, domestic and foreign efforts are devoted to the research of new grain refiners including Al-Ti-C, Al-Ti-C-B, Al-Ti-B-Re, and Al-Ti-C-Re. The addition of C and Re can greatly improve the precipitation and aggregation of TiAl_3_ and TiB_2_ and increase the viscosity of aluminum melt. The addition of C can not only avoid the pollution of the environment by harmful gases in the production process but also eliminate the inclusion generated by the reaction with salt. The addition of Re can improve the decay delay of Al-Ti-B and greatly improve the thinning efficiency. However, due to the poor wettability of C and the alloy matrix, the yield of C is low, which reduces the production efficiency. Furthermore, Re is expensive, and the preparation process is complex. So, it is still necessary to develop more efficient process technology to achieve large-scale industrial production.

### 2.2. Al-Ti-C

The refining capacity of Al-Ti-carbon grain refiner is lower than that of Al-Ti-boron grain refiner, and it has obvious attenuation. Coupled with the complex preparation process, Al-Ti-C is a terpolymer intermediate alloy refiner that was proposed before Al-Ti-B. In the 1950s, the carbide and boride nucleation theory proposed by Cibula [58] suggested that TiB_2_ and TiC were good heterogeneous nucleation bases for α-Al. TiB_2_ and TiC particles with a high melting point can still exist stably in aluminum melt after being melted by Al-Ti-B and Al-Ti-C grain refiners and become heterogeneous nucleation substrates during α-Al solidification. Subsequently, Cibula tried to prepare Al-Ti-C intermediate alloy by various methods but was not successful due to the poor wettability of carbon in aluminum liquid and the small density of carbon that floated easily in aluminum liquid. In 1985, Banerji obtained Al-Ti-C intermediate alloy using the strong stirring method in the laboratory. Since then, Al-Ti-C intermediate alloys have once again attracted wide interest [9].

Al-Ti-B is widely used as a grain refiner for aluminum alloy, but TiB_2_ particles interact easily with Zr, Mn, Cr, and other elements in aluminum alloy, resulting in the “refiner poisoning” phenomenon, which leads to the decline of its refining effect, causing uneven grain structure. In addition, in Al-Ti-B refiner, TiB_2_ particles, which can be used as an effective nucleating core, agglomerate and precipitate easily, which will damage the surface quality of rolled sheet and foil [59,60,61]. TiB_2_ particles are usually less than 1% of the total, which means the refiner’s nucleation ability is limited, so it is necessary to add a lot of Al-Ti-B refiner to reduce the grain size of aluminum alloy to 100 μm. Al-Ti-C grain refiner has an excellent grain-refining effect [50,62]. TiC is the main heterogeneous nucleation particle and plays the role of refining α-Al grains, and TiAl_3_, as a good flux, can promote the wetting of carbide and aluminum melt, thus promoting the formation of TiC, providing conditions for refining. TiC particles are much smaller than TiB_2_ particles, with an average size of about one-third that of TiB_2_ particles. The small size of TiC particles makes TiC in aluminum melt difficult to precipitate to the bottom of the melt, which makes Al-Ti-C grain refiner have good resistance to refining decline [63]. In addition to the advantages of TiC particles, Al-Ti-C grain refiner has a certain degree of resistance to Si and Zr poisoning, unlike Al-Ti-B grain refiner, which loses its refining ability in the case of Si and Zr (only 0.2 wt.%). Zr can eliminate the refining effect of Al-Ti-B in a short time [64,65]. TiC particles are too stable in liquid aluminum to react with alloying elements such as Mn, Cr, and Zr, so TiC particles are immune to the phenomenon of “poisoning” of elements such as Zr, Cr, V, Si, and Mn. The aggregation tendency of TiC particles is smaller than that of TiB_2_ particles. Al-Ti-C intermediate alloys contain a large number of TiAl_3_ and TiC phases and are currently the best choice to replace Al-Ti-B refiners [66].

**Figure 3:**
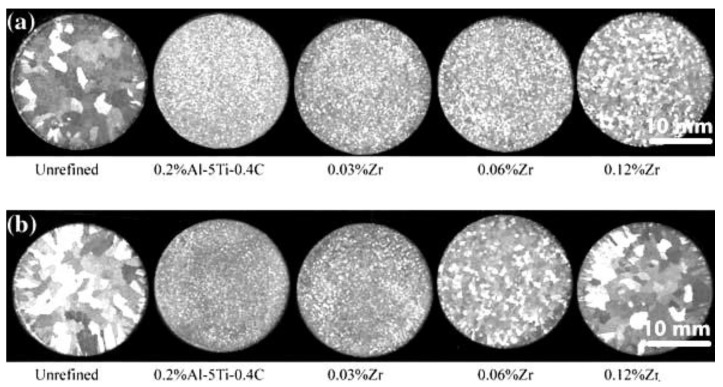
Macrostructures of samples refined with 0.2%Al–5Ti–0.4C for different content of Zr. (**a**) The commercial purity Al melted at 730 °C; (**b**) The commercial purity Al melted at 800 °C [63].

Al-Ti-C series grain refiners avoid Zr poisoning. It is generally proposed that TiC particles in Al-Ti-C grain refiners have a smaller aggregation tendency than TiB_2_ particles in Al-Ti-B and have toxic “immunity” to Zr, Cr, Si, V, and Mn [63]. However, Ding showed that Zr could also poison Al-Ti-C grain refiner [48]. Studies have shown that the Zr poisoning of Al-Ti-B and Al-Ti-C grain refiners or the degree of weakening or disappearance of their refining effect is related to many factors such as melting holding time and temperature [67]. The effect of Zr on the grain refinement of the commercial purity Al is shown in Figure 3. It can be observed that at 730 °C, the grain coarsens gradually with increasing Zr content. The poisoning is mainly due to the reaction between the Zr-containing phase in aluminum alloy and the Al_3_Ti phase or related phase (TiB_2_/TiC) in the refiner [60,68,69,70]. And the substitution of Zr elements on the surface of TiB_2_/TiC particles forms ZrB_2_/ZrC [49]. These two theories are affected by the low content, wide distribution, or unequal distribution of Zr elements or grain refiners in the alloy, and it is difficult to observe the phenomenon whereby the Zr-containing phase reacts with the related phase in the refiner and forms a composite phase. There is no direct evidence to support either theory.

At present, the main preparation methods are the solid–liquid reaction method, reaction sintering method, SHS (self-propagating high-temperature synthesis) casting method, XD (exothermic reaction) method, VLS (gas–liquid–solid contact reaction) method, etc. In the 1980s, Banerji and Reif prepared Al-Ti-C grain refiner by adding preheated graphite powder to superheated Al-Ti melt using the solid–liquid reaction method [9,11]. The SHS method mixes the matrix metal powder with the solid–liquid powder containing the internal phase components and ignites the sample in the test device with the ignition tungsten wire so that the chemical reaction between Al, Ti, and C is generated and the combustion wave is spread, and when the combustion wave is pushed forward, the reactant becomes the Al-Ti-C refiner. The XD method mixes both the solid reactive element powder and metal substrate powder evenly after compacting and degassing, pressing the block under vacuum conditions, and rapidly heating to the temperature above the melting point of the metal substrate. In the metal melt medium, the two solid reaction elements diffuse with each other, contact, and constantly react to precipitate a stable reinforcement phase, and then the melt is cast and extruded. The VLS method was invented by Koczak in 1989. The VLS process mainly includes two reaction processes: first, under the action of the high-temperature alloy liquid, the gas in the alloy liquid is dispersed. The second is the chemical reaction of a gas or element obtained after decomposition in the alloy liquid.

The preparation technology of Al-Ti-C refiner is seriously lagging and still has not been applied on a large scale, mainly because of the poor wettability of liquid aluminum to carbon during the preparation process, which makes alloying difficult and makes it difficult to realize industrial production and practical application. At 1000 °C, the wetting angle between graphite and liquid aluminum is still greater than 90°, which is a nonwetting state. Improving wettability has become a key technology for preparing Al-Ti-C grain refiners.

### 2.3. Al-Ti-B-C

Al-Ti-B and Al-Ti-C series intermediate alloys have been widely used in the grain-refining process of aluminum alloy, and the refining effect is obvious. The main reason is that there is excellent nucleation base TiB_2_ or TiC in the refiner, and the solute Ti can significantly inhibit the grain growth of Al. However, the agglomeration effect of TiB_2_ particles in Al-Ti-B intermediate alloy can cause pinhole defects in aluminum foil and strip defects in anodized sheet. In addition, Zr, Cr, Si, and other elements will also lead to TiB_2_ surface “poisoning”, so that the refining performance rapidly declines. These disadvantages limit the application of Al-Ti-B intermediate alloys. Al-Ti-C series intermediate alloy is considered to be an ideal substitute for it. However, it was found that the long-term thermal insulation treatment in the refining process may cause the decomposition of TiC particles, thus reducing the refining performance of the intermediate alloy. In addition, ternary intermediate alloys are often produced by the fluorine salt method in the process of preparation, but the production process has environmental problems. Moreover, the refining effect of the traditional Al-Ti-B series intermediate alloy on Al alloy has reached the limit, and the question of how to break this refining bottleneck has become an important topic. A series of Al-Ti-B-C intermediate alloys were prepared by reacting liquid aluminum with pure Ti, graphite powder, and Al-3B intermediate alloys. The results show that Al-Ti-B-C quaternary intermediate alloys containing TiAl_3_, TiB_2_, and TiC particles can synthesize the advantages of Al-Ti-B and Al-Ti-C intermediate alloys. Nie avoids the poisoning phenomenon of TiB_2_ in aluminum alloys containing Cr, Zr, and Mn and the decline of the anti-decay phenomenon caused by the reaction of TiC particles with aluminum melts, obtaining better refining properties [71].

Compared with Al-Ti-B and Al-Ti-C intermediate alloys, Al-Ti-B-C has stronger resistance to Zr “poisoning”. Both Al-Ti alloy and Al-Zr alloy have a good refining effect when refining aluminum alloy alone, but when they are added to alloy melt at the same time, the grain-refining performance will be significantly weakened; that is, the two appear to produce a mutual “poisoning” phenomenon [46]. Al-Ti-B-C quaternary intermediate alloys containing TiAl_3_, TiB_2_, and TiC phases have excellent refining properties.

The wettability of Al and B_4_C is the key to the preparation of Al-Ti-B-C. Some studies consider that in a vacuum state of 1200 °C and 1300 °C for 15 min, the contact angle of Al/B_4_C is 45° and 20°, respectively, but with the addition of 10% Ti element, the contact angle under the same state is rapidly reduced to 0°. High-energy ultrasonic treatment and induction melting can change the local or whole reaction temperature of the melt, which has potential positive effects on improving the wettability of Al/B_4_C and promoting the reaction process. While meeting the requirements of grain refinement, it can effectively avoid the pollution and disposal problems of harmful waste residue in the traditional process.

### 2.4. Al-Ti-B-(C)-Ce

With special electron layer structure and active chemical properties, rare-earth elements have the reputation of being “industrial vitamins”. They can form rare-earth compounds with almost all kinds of elements. Rare earth can not only play a refining and metamorphic role in aluminum alloy but can also remove gas and impurities, reduce the pinhole rate, and play a refining role. Rare earth is the only alloying element that can improve the corrosion resistance of aluminum and its alloys. Among the alloys, Al-Ti-C, Al-Ti-B, and other intermediate alloys are refiners used for aluminum and aluminum alloys, and remarkable results have been achieved. However, the nucleation particles of these refiners are often obtained by in situ reaction, and their morphology, size, and aggregation state are difficult to control, which increases the difficulty of regulation. Rare earth can improve the morphology and distribution of the second-phase particles in Al-Ti-B or Al-Ti-C refiners, thereby increasing their refining effect. Therefore, rare-earth composite refinement is regarded as an important direction for grain refinement of aluminum alloys in the future.

The advantages of rare earths as refining agents are currently divided into two theories. One is that in the high melting point compounds formed by rare-earth elements and metallic aluminum, certain alloying elements or impurities (Fe, Si, S, etc.) act as heterogeneous nuclei and precipitate at the grain boundaries. Due to the pinning effect and organization of grain growth, the high surface activity of rare earths can reduce the surface energy of the nucleus and increase the rate of crystalline nucleation. The other is that due to the low solid solubility of rare-earth elements in aluminum melt and of surface active substances, it is easy to adsorb the polarization of grain boundaries and phase surfaces and fill the defects on the interface, thus hindering the growth of nucleation cores. The lattice mismatch between Ce and Al is 27.3%, which means that they are insoluble in Al and do not form a replacement solid solution with Al. Owing to its high surface energy, the aluminum melt is wrapped on the surface of the aluminum grain during crystallization, which hinders the growth of the grain. Rare earth and aluminum form a compound with a high melting point, which stably exists in aluminum melt as the core of heterogeneous nucleation, hindering the growth of grains and refining the grains of the matrix. In addition, the existence of rare-earth phase distributed at the grain boundary forms a compositional supercooling mechanism to metamorphize the aluminum melt and refine the grain size. Adding trace rare earth to high purity aluminum has the effect of refining pure aluminum grains. Adding about 0.3% aluminum–cerium alloy refiner can achieve the best refining effect. Al_11_Ce_3_ and α-Al produced by eutectic reaction during the preparation of Al-Ce intermediate alloy have similar crystal structure, and the lattice constant can be corresponding, so Al_11_Ce_3_ can be used as the heterogeneous nucleation point during the solidification of α-Al, thus promoting thinning. By refining the pure aluminum grains, the pure aluminum materials can be strengthened and toughened. The mechanism of rare-earth refining of pure aluminum affects the solute distribution within the grain and at the liquid–solid interface.

**Figure 4:**
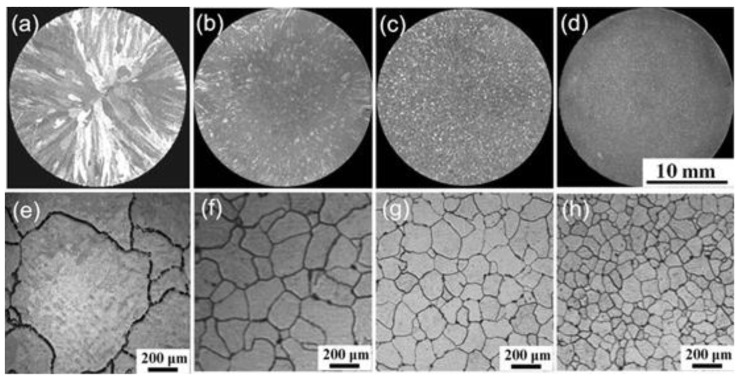
Optical images of as-cast aluminum samples: (**a**,**e**) pure Al without inoculation; (**b**–**d**,**f**–**h**) are inoculation with in situ CeB_6_/Al composite inoculant with different cooling rates, respectively [72].

An Al-B-Ce intermediate alloy containing Al, CeB_6_, and Al_11_Ce_3_ phases was prepared by vacuum arc furnace. The particle size of CeB_6_ was detected to be about 2–4 μm and was found in clusters in the aluminum matrix. Al-B-Ce intermediate alloy was rapidly solidified to prepare a thin strip of CeB_6_/Al inoculant. As shown in Figure 4, CeB_6_/Al with different cooling rates has different inoculation effects. The results showed that compared with Al-B-Ce intermediate alloy, CeB_6_ particles became smaller and dispersed in the aluminum matrix. The enhancement effect of CeB_6_/Al inoculant depends on the size of CeB_6_ phase. The thin strip CeB_6_/Al inoculant, with an average particle size of about 500 nm, has a better refining and strengthening effect. According to the calculation of mismatch theory, the mismatch between CeB_6_ particles and Al is small and the lattice match is good. Therefore, CeB_6_ particles can be used as an effective and stable nucleation core of Al.

The addition of rare earth has a significant effect on the size, morphology, and distribution of TiAl_3_ phase of Al-Ti-B intermediate alloy, and the addition of an appropriate amount of rare earth can reduce the wetting angle between Al melt and TiB_2_, make TiB_2_ difficult to aggregate and precipitate, and enhance the refining property of the intermediate alloy. Therefore, the combination of rare earth and Al-Ti-B is regarded as an effective method to improve the refining effect of aluminum, titanium, and boron.

The rare-earth element Ce can improve the wettability between graphite and liquid aluminum and solve the key problem of the wettability of Al-Ti-C series grain refiners. The surface activity and catalytic action of rare-earth element Ce effectively improves the wettability between graphite and liquid aluminum, promotes the reaction of graphite, and increases the amount of TiC production. Rare-earth and Al-Ti-C composite thinning TiAl_3_ phase not only maintain blocky and uniform distribution but also reduce the agglomeration of TiB_2_ particles, resulting in an enhanced thinning effect. For example, it has an “immune” effect on Mn, Cr, Zr, and other elements in aluminum alloy, is difficult to decompose, and is difficult to aggregate with TiC particles. However, Al-Ti-C intermediate alloy research started late, so the refining effect is unstable, and the wettability between aluminum liquid and carbon is poor, which seriously limits the application of Al-Ti-C intermediate alloy in actual production. Ding studied the effect of rare-earth element Ce on the microstructure and refining effect of Al-5Ti-0.6C-1Ce grain refiner [73]. Rare-earth Ce improves the refining effect of Al-5Ti-0.6C-1Ce grain refiner because it promotes the formation of TiC particles, but it has a great influence on the synthesis of TiC particles; Xu prepared Al-Ti-C-La grain refiner containing La using self-propagating high-temperature technology, and found that La can improve the wettability of liquid aluminum and graphite, and the addition of La leads to the dispersion of TiAl_3_ and TiC particles in the aluminum matrix. The rare-earth element Ce was introduced into Al-5Ti-0.25C grain refiner, the wettability between graphite and liquid aluminum was improved by the surface activity and catalysis of rare-earth element, and the refining effect was improved. The microstructure of Al-5Ti-0.25C-1Ce grain refiner not only contains more TiAl_3_ and Ti_2_Al_20_Ce phases, but also ensures that the phase size of TiAl_3_ and Ti_2_Al_20_Ce is not too large. The contents of TiAl_3_ and TiC are 44.52% and 60.87%, respectively, higher than those of Al-5Ti-0.25C grain refiner.

Two different forms of Al-Ti-B-C-Ce inoculant were prepared by vacuum arc furnace and vacuum rapid quenching furnace. The reinforced particle size of Al-Ti-B-C-Ce intermediate alloy can reach several microns. It is then quickly melted and cooled to obtain Al-Ti-B-C-Ce inoculant ribbons in which the size of the reinforcing particles is about a few hundred nanometers. Compared with the intermediate alloy, the phase composition of the inoculant ribbon did not change, but the reinforcement particles became smaller, with a size of about 600 nm, and dispersed in the Al matrix. Due to the similarity of crystal structure, Al_3_Ti and Al have a good lattice matching relationship. The addition of rare-earth elements promoted the formation of Ti_2_Al_20_Ce phase. The presence of rare earth not only plays the role of dehydrogenation, deoxygenation, and purification of melt, but also changes the morphology of Al_3_Ti from acicular to massive and becomes a reinforcement that does not split the matrix and is dispersed in the Al matrix, which not only increases the dispersion strengthening effect, but also improves the grain-refinement effect.

Song conducted a study on adding (Pr + Ce) rare-earth mixture to Al-7Si-0.7Mg alloy and added 0.6 wt% (Pr + Ce) rare-earth mixture to obtain the best refining effect. The best grain-refining effect can be obtained by adding different contents of La and Ce composite rare earths to La and Ce alloys to form a variety of compounds with other alloying elements. These compounds can be used as heterogeneous nucleation substrate to improve the nucleation rate of alloys. At the same time, La and Ce are enriched at the front of the solid–liquid interface, which hinders the growth of α-Al.

### 2.5. Al-Sc Alloy

Scandium (Sc) is the most effective alloying element for aluminum alloy found so far. It can significantly improve the properties of aluminum alloy. Sc is not only a rare-earth element but also a transition metal element, which has the advantages of both. Sc can not only refine the aluminum alloy structure but also increase the recrystallization temperature and hinder grain growth, thereby improving the strength of the material, corrosion resistance, and heat resistance. The grain-refining effect of scandium is due to the fact that the intermetallic compound Al_3_Sc and α-Al have the same FCC (face-centered cubic) structure. Its lattice constant is about 0.414 nm, while that of α-Al is 0.412 nm at 659 °C [74]. At this time, the mismatch is only about 0.5%, and the interface energy and distortion energy needed to be overcome by the nucleation of aluminum atoms at the Al_3_Sc interface are small, which plays a very effective role in the nucleation core. The Al_3_Sc precipitated from the solid solution is dispersed in fine particles, which can significantly improve the strength and recrystallization temperature of aluminum alloy semifinished products.

**Figure 5:**
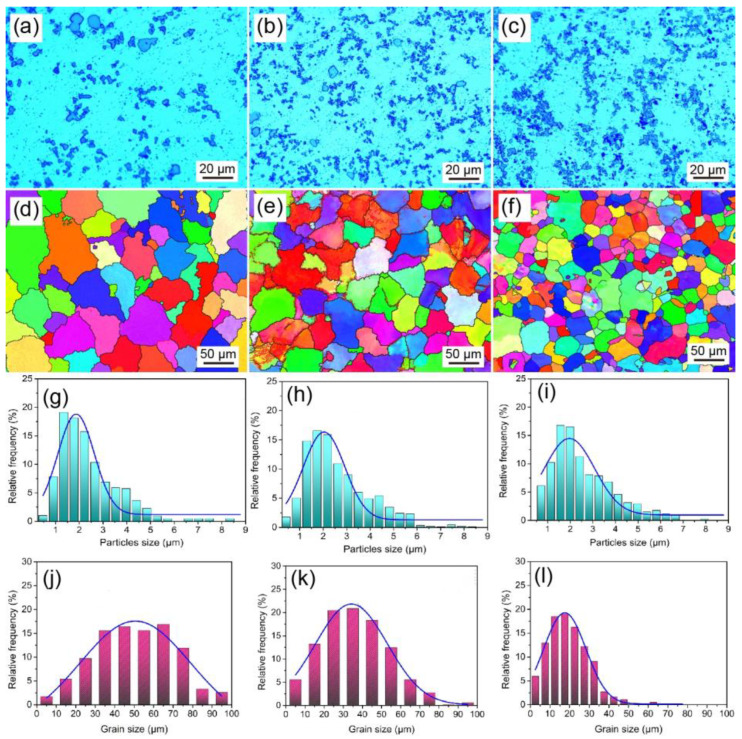
(**a**–**c**) Representative OM images of as-cast Al-xSc (x = 2, 4, 8) alloys, with corresponding EBSD maps of (**d**–**f**). (**g**–**i**) are the particle size distribution maps of as-cast Al-xSc alloys. (**j**–**l**) are the statistical distribution of grain size of as-cast Al-xSc alloys [74].

The grain-refinement effect of Sc and Ti combined in aluminum alloy is better than that of a single addition. Sc has an excellent grain-refining effect on Al alloy and can improve the as-cast microstructure of Al alloy. Figure 5 shows the optical image, EBSD image, and statistical analysis results of the as-cast Al-Sc alloy. It can be seen that the grain size decreases with the increase in Sc content. Without changing the grain size, Sc content has a significant effect on the number of grains. Li found that with an increase in Sc content, not only the as-cast grains of an Al-Zn-Mg-Mn alloy were refined, but also the microstructure gradually changed from dendrite to equiaxed. It is worth mentioning that a semicontinuous ingot of Al-Mg-Mn-Zr aluminum alloy containing Sc has a uniform equiaxed crystal structure, and the average grain size is only about 22 μm [75].

Sc and Zr added to aluminum at the same time will also produce an excellent grain-refinement effect [76]. The mechanism of Sc and Zr composite grain refinement is that the generated Al_3_Zr and Al_3_Sc phases have a lattice structure similar to α-Al and can be used as nucleation particles, which increases the nucleation rate of the alloy. The combination of rare-earth and non-rare-earth elements can reduce the amount of rare-earth metals and thus reduce costs. Yin studied the effect of the composite effect of rare-earth Sc and Zr on the microstructure and mechanical properties of Al-Mg alloy. The results show that the grain size of Al-5Mg alloy is reduced from 370 μm to 42 μm, and the tensile strength is increased from 260 MPa to 398 MPa. At the same time, the elongation of 18% is maintained. It is found that the as-cast grain of Al-Zn-Mg-Cu alloy can be significantly refined by adding trace Sc and Zr, and the dendrite structure can be reduced. The average grain of Al-Zn-Mg-Cu alloy can be refined to 35 μm. In summary, the advantages and disadvantages of different types of inoculants are shown in Table 1.

### 2.6. Fe-Rich Phase in Al-Si Alloy

In conventional cast Al-Si alloys, the primary α(Al) phase in the room temperature solidification structure is usually dendritic with good strength and toughness, while the Si phase is hard and brittle. The primary Si is generally a polygonal block, and the eutectic Si is mostly of a long acicular or lamellar structure. Al-Si alloys with lower Si content usually have excellent casting properties, fatigue resistance, and corrosion resistance [45]. With the increase in Si content, when eutectic silicon and primary silicon are precipitated in large quantities, the Al matrix will be cut, and the ductile strength of the alloy will be seriously reduced. In industrial production, Al-Si alloys with a Si mass fraction greater than 6% are usually modified. After modification treatment, the primary Si will become fine and rounded from a large polygonal block, and the eutectic silicon will become fine lamellar, or even fibrous, from sharp, long needles. The transformation of the Si phase microstructure greatly promotes the improvement of the mechanical properties of the alloy. Modification treatment has become the most extensive and effective means to improve the properties of Al-Si alloys. Subeutectic and eutectic Al-Si alloys are usually modified by chemical modification, rapid solidification, ultrasonic treatment, superheating treatment, melt stirring (including mechanical stirring and electromagnetic stirring), melt pretreatment, current, or electric field treatment. The metamorphic mechanism lies in controlling the nucleation and growth of eutectic Si [45]. At present, the chemical metamorphism method is mainly used in industry, and the addition of the metamorphic agent method has the advantages of stable metamorphic effect, strong operability, mature process, and no additional equipment.

The earliest chemical modifier used for hypoeutectic and eutectic Al-Si alloys was metal Na, but the low boiling point and overactive chemical properties of metal Na caused a lot of inconvenience to the production. Because of its small density, the modified alloy structure showed obvious specific gravity segregation and, generally, has been replaced by sodium salt metamorphism. At present, binary (67 wt.% NaF + 33 wt.% NaCl) or ternary (25 wt.% NaF + 62 wt.% NaCl + 13 wt.% KCl) sodium salt mixtures containing NaF are mainly used in industry as eutectic silicon metamorphisms. The advantages of this method are low application cost, good metamorphic effect, no latency, and insensitivity to cooling rate. Its disadvantages are short effective metamorphic time, remelting failure, and corrosion of crucible equipment, which will cause certain harm to the environment and human body. The addition of Sb could change the morphology of eutectic Si from needle-like to rod-like, but it was eliminated due to its high cost and toxic properties. With the deepening of the study of metamorphic agents, many countries began to use Sr metamorphic instead of Na. The metamorphic effect of Sr is similar to that of Na, and eutectic Si can also be refined into fiber [77]. Sr modification is usually added in the form of Al-10Sr intermediate alloy, and the addition of Sr near 0.02% can obtain a good metamorphic effect. Sr deterioration has many advantages such as its 5~7 h long-term performance, good remelting, small dosage, and production that is easy to control. Furthermore, it does not cause excessive deterioration, does not pollute the environment, does not corrode equipment, etc. Its disadvantages are that Sr is easy to oxidize and burn, it increases the alloy suction tendency, and its deterioration has a latency of about 40 min.

Sr has a good metamorphic effect on eutectic Si and promotes the nucleation growth of coarse columnar or dendritic α-Al grains, which indicates that it is not enough to only degrade eutectic Si for casting Al-Si alloys, and it is necessary to equiaxial and fine the α-Al dendrites to eliminate the adverse effects of this microstructure on the mechanical properties of the alloys.

Al-Ti-C and Al-Sr are added at the same time. The two do not interfere with each other, and when Al-Ti-C refines α-Al, it can promote the metamorphism of eutectic Si to a certain extent. This is because during the solidification and growth of Al-Si alloy, α-Al dendrites are precipitated, first, and then grown during the subsequent eutectic transformation. Eutectic Si is precipitated and grown in the gap between α-Al dendrites. Therefore, the size of the eutectic Si must depend on the size of the α-Al dendrites. Thus, while refining α-Al dendrites, it is shown that refining treatment has a certain metamorphic effect on eutectic Si. This mutual promotion of refinement and metamorphism can reduce the amount of modifier or refiner added, to a certain extent, and reduce production costs when the same refinement and metamorphism are obtained.

**Figure 6:**
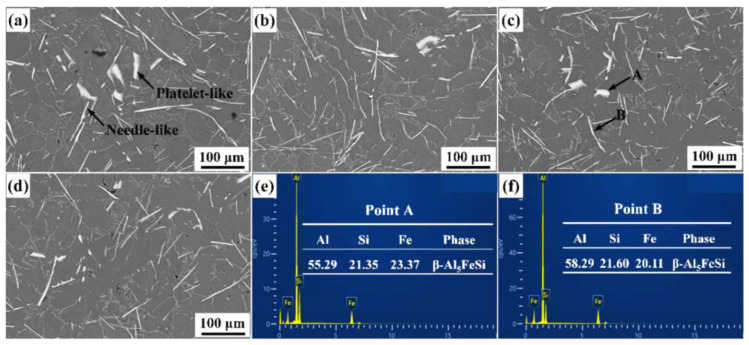
The morphology of the Fe-rich phases with different B contents ((**a**) B00 alloy; (**b**) B15 alloy; (**c**) B30 alloy; and (**d**) B60 alloy), and chemical compositions at Point A (**e**) and B (**f**) [89].

Iron is an unavoidable impurity in Al-Si alloys and has long been regarded as a harmful element. These iron impurities are originally derived from electrolytic brocade and are continuously increased in the later continuous melting process through the corrosion of the cauldron and various tools. Additional iron impurities are caused by incomplete sorting in the recycling process. Since the solid solubility of Fe in aluminum and aluminum alloys is only 0.05%, Fe basically exists in the form of iron-rich phase in aluminum, such as AlFe_3_, Al_5_FeSi, Al_8_Fe_2_Si (MnFe)Al_6_, (CuFe)Al_6_, etc. [78]. The iron impurities in the alloy can form a variety of complex lamellar intermetallic chemicals with other elements, resulting in serious separation of the matrix [45]. In addition, the large size of the iron-rich phase has an adverse effect on the feeding of the alloy after solidification, thereby increasing the formation tendency of porosity, so the iron content is often required to be low in the cast alloy to avoid the deterioration of its mechanical properties [79,80,81]. When the iron content is high, many methods can be adopted to change the shape of the iron-rich phase or limit its size to reduce its harm. Metal elements can usually be added to transform the iron-rich phase from a sheet-like phase to a kanji-like quaternary or multicomponent iron-rich phase [82,83]. However, the addition of these elements leads to a further increase in the content of the iron-rich phase, which makes them impossible to use without restriction [84,85]. In addition, the use of high cooling speed, ultrasonic treatment, friction stir processing, and other methods can limit the size of the iron-rich phase and can transform the iron-rich phase of the crystal type and improve the mechanical properties of the alloy [86,87,88]. As shown in Figure 6, the optical micrographs of Al-Si-Fe at different cooling rates show different stages of iron-rich phase [89].

The common method of removing iron is to form the first precipitated compound with the iron element and other additives, and then use filtration, centrifugation, electromagnetic separation, precipitation, and other methods to separate these compounds. Electrolysis methods were invented by Hoopes, but these methods have some problems; e.g., they are time-consuming and have a low impurity removal efficiency. Recently, the newly developed electroslag remelting method has obtained good iron removal efficiency. This method uses a slagging agent to react with iron to form an iron–boron intermediate compound during the electroslag remelting process, which is then captured by the molten slag agent [90,91].

## 3. Conclusions

(1) In recent years, efforts have been made to develop new efficient and environmentally friendly grain refiners for aluminum alloys, including Al-Ti-C, Al-Ti-C-B, and Al-Ti-B-C-Ce, and adding rare-earth elements. The addition of Re element can improve the decay delay of Al-Ti-B and greatly improve the refining efficiency. However, due to the poor wettability of C and the Al matrix, the yield of C is low and the production efficiency is reduced, while Re is expensive and easy to oxidize, and more efficient process technology still needs to be developed to achieve large-scale industrial production.
(2) Sc is the most effective element for grain refinement of aluminum alloy, and its mismatch with aluminum matrix is only 0.5%. The interface energy and distortion energy needed to be overcome by the nucleation of aluminum atoms at the Al3Sc interface are very small, and it plays a very effective role in nucleation. However, its application as nucleation substrata in production requires further consideration of cost and environmental issues. Therefore, Sc acts together with other elements, such as Ti and Zr, which is a very promising research direction for the future.
(3) The excessive iron element in Al-Si alloy is the key problem restricting its mechanical properties and recycling. Whether it is chemical modification, rapid solidification, ultrasonic treatment, or overheating treatment, there are complex processes and environmentally unfriendly problems; therefore, Al-Si alloy will develop in the direction of high efficiency, low addition, high purity, and high stability, and the research work of the grain refinement mechanism will be gradually advanced.

## Figures and Tables

**Table 1:** Disadvantages and advantages of different kinds of inoculants.

Refiner Type	Obvious Advantage	Analysis of Disadvantages
Al-Ti-B	High refining efficiency: 12 times higher than Al-Ti Al3Ti and TiB2 are excellent nucleation cores of Al [40,41] TiB2 particle size is 4~5 μm [42] The addition of 1.5 wt.%~2.5 wt.% has an effective grain-refinement effect	TiB_2_ is easy to accumulate and deposit, causing quality problems [43] In the case of elements such as Zr, Cr and Mn, it will be “poisoned” and lead to failure [43]
Al-Ti-C	Pure and pollution-free Immune to elements such as Zr, Cr, and Mn [63] TiC particle size is 2~3 μm. TiC particle size is smaller [62,65]	Poor anti-attenuation performance [58] The refinement effect is slightly poor [58] C has poor wettability with matrix [9]
Al-Ti-B-C	Refining efficiency has been further improved [46] Strong wettability with matrix Immune to elements such as Zr, Cr, and Mn [71]	Borides tend to segregate [43]. Carbide stability is poor
Al-Ti-B(C)-Ce	Includes the advantages of the above three refiners [73] Effective control of particle size, morphology, and aggregation state [72] The grain size of industrial pure aluminum can be refined from 1430 μm to 227 μm by adding 0.3 wt.%	Moderate dosage control High temperature preparation can lead to oxidation caking and other problems
Al-Sc alloy	Has the advantages of both rare-earth elements and transition metals [74] Adding 0.1~0.2% Sc can increase the recrystallization temperature of the alloy by 150~220 °C The addition of 0.05~0.1 wt.% has an effective grain-refinement effect [75]	Resources are scarce, dispersed and expensive Dosage control. The performance deteriorates if the Sc content exceeds 4%

## Data Availability

Not applicable.
